# Deep Learning System Boosts Radiologist Detection of Intracranial Hemorrhage

**DOI:** 10.7759/cureus.30264

**Published:** 2022-10-13

**Authors:** Roshan Warman, Anmol Warman, Pranav Warman, Andrew Degnan, Johan Blickman, Varun Chowdhary, Dev Dash, Rohit Sangal, Jason Vadhan, Tulio Bueso, Thomas Windisch, Gabriel Neves

**Affiliations:** 1 Neuroscience, Caire Health, Inc., Tampa, USA; 2 Radiology, University of Pittsburgh Medical Center (UPMC) Children’s Hospital of Pittsburgh, Pittsburgh, USA; 3 Radiology, Caire Health, Inc., Tampa, USA; 4 Emergency Medicine, Stanford University, Stanford, USA; 5 Emergency Medicine, Yale School of Medicine, New Haven, USA; 6 Emergency Medicine, The University of Texas Southwestern (UTSW), Dallas, USA; 7 Neurology, The Texas Tech University Health Sciences Center (TTUHSC), Lubbock, USA; 8 Radiology, Covenant Health, Lubbock, USA

**Keywords:** radiologist, intracranial hemorrhage, diagnosis, deep learning system, artificial intelligence

## Abstract

Background: Intracranial hemorrhage (ICH) requires emergent medical treatment for positive outcomes. While previous artificial intelligence (AI) solutions achieved rapid diagnostics, none were shown to improve the performance of radiologists in detecting ICHs. Here, we show that the Caire ICH artificial intelligence system enhances a radiologist's ICH diagnosis performance.

Methods: A dataset of non-contrast-enhanced axial cranial computed tomography (CT) scans (n=532) were labeled for the presence or absence of an ICH. If an ICH was detected, its ICH subtype was identified. After a washout period, the three radiologists reviewed the same dataset with the assistance of the Caire ICH system. Performance was measured with respect to reader agreement, accuracy, sensitivity, and specificity when compared to the ground truth, defined as reader consensus.

Results: Caire ICH improved the inter-reader agreement on average by 5.76% in a dataset with an ICH prevalence of 74.3%. Further, radiologists using Caire ICH detected an average of 18 more ICHs and significantly increased their accuracy by 6.15%, their sensitivity by 4.6%, and their specificity by 10.62%. The Caire ICH system also improved the radiologist’s ability to accurately identify the ICH subtypes present.

Conclusion: The Caire ICH device significantly improves the performance of a cohort of radiologists. Such a device has the potential to be a tool that can improve patient outcomes and reduce misdiagnosis of ICH.

## Introduction

There were an estimated 23 million head CT scans performed in 2022 in the United States, a figure that has consistently grown over the past few decades [1]. Its short acquisition time, widespread availability, and ever-decreasing radiation burden, have made the head CT a staple of care worldwide, particularly in the emergent setting [2]. However, increased head CT utilization has not coincided with an increase in the supply of qualified radiologists available to read the acquired scans. This shortage of image interpreters leads to increased per-radiologist volume, increased fatigue, and burnout [3]. Factors like increased workload and fatigue are well understood to contribute to human error in radiologist interpretation [3-5].

An erroneous diagnosis is always damaging, but principally for acute pathologies where time plays a critical role in patient outcomes. Acute intracranial hemorrhage (ICH) generally appears hyperdense compared to the surrounding brain parenchyma and may require urgent intervention. Intracranial hemorrhage is one of many conditions that can be readily diagnosed from a head CT and has a poor prognosis with only 20% of survivors fully recovering at six-months post-incidence [6-8]. Nonetheless, there is expert consensus that rapid response and treatment of ICH may significantly improve patient outcomes [8].

Artificial intelligence (AI) technologies, specifically computer vision (CV) algorithms, have continued to expand their applications to the interpretation of various medical imaging modalities, given their ability to rapidly interpret these scans in coordination with their demonstrated strong performance [9,10]. Despite evidence suggesting that a concerted effort by AI and clinicians can yield higher diagnostic accuracy than either party alone, particularly for inexperienced practitioners, the role of an AI tool as an adjunct to radiologists to reduce human diagnostic error with respect to ICH is not yet widely adopted [11]. Additionally, though algorithms have been constructed to evaluate scans for the presence of an ICH, they treat ICH as a homogenous pathology, when in fact various subtypes of ICH are observed and often managed in differing ways [12-15].

In this study, we evaluate the utility of a novel deep learning algorithm designed to aid radiologist diagnostic performance by identifying the presence of ICH and its respective subtype(s) on a non-contrast head CT scan (NCCT).

## Materials and methods

Ethical approval and reporting guidelines

As the study involved the secondary analysis of previously collected data, it is IRB-exempt. This study followed the Transparent Reporting of a Multivariable Prediction Model for Individual Prognosis or Diagnosis (TRIPOD) guidelines.

Data source 

We obtained 600 NCCT scans of the head and their radiological impressions from Segmed, Inc. (Stanford, CA). We specifically obtained 100 ICH-negative NCCTs and 100 individual studies for each ICH subtype: epidural (EDH), subdural (SDH), subarachnoid (SAH), intraparenchymal (IPH), and intraventricular hemorrhage (IVH). We allowed scans to have multiple subtypes of hemorrhage present at one time.

All NCCT head scans had protected health information (excluding age and sex) removed from the reports and DICOM tags. We obtained the included cases from inpatient, outpatient, and emergency settings. We filtered these scans to ensure (1) patients were greater than 18 years old; (2) the scans were unenhanced; (3) the scans were devoid of motion artifact; (4) the scans' slice thickness was >1.5 mm; (5) scans were obtained using a standard convolutional kernel; and (6) scans were in an axial projection.

Study design and participants

This retrospective, multi-reader, and multi-case study evaluated the diagnostic accuracy of three board-certified radiologists with and without the aid of a deep-learning system. The radiologists had an average of 13 years of experience post-residency (2, 3, and 34 years), all of whom were board-certified in Diagnostic Radiology by the American Board of Radiology. Each radiologist interpreted each imaging case independently. After a washout period of 48 hours, the radiologists interpreted the same cases with the support of the deep learning system. We used the MD.ai (MD.ai, Inc., New York) platform to view and label each NCCT scan, and scans were presented in a sequentially different manner in each review session. The platform provided the option for readers to modulate windowing, both manually or with presets, and offered an array of annotation and measurement tools.

Before any labeling of NCCT scans, each radiologist underwent training and a screening examination to assess: (1) their capacity to appropriately view the scans on the MD.ai platform; (2) their capacity to accurately use the labeling tools of the MD.ai platform; and (3) their capacity to interpret the outputs of the deep learning model through the MD.ai platform.

Labels and ground truth determination

Each radiologist labeled a scan independently for the presence or absence of an ICH. If an ICH was suspected, the radiologist had to specify which subtypes (EDH, SDH, SAH, IPH, and IVH) were present. We obtained ground truth values for each scan through a consensus of the three radiologists in a manner consistent with prior studies [16,17]. When a unanimous consensus was not achieved, we established the ground truth from a unanimous or majority (2 of a panel of 3) consensus.

Deep learning algorithm and performance assessment

The radiologists used the Caire ICH (Caire Health, Inc., Tampa, FL) at its predetermined operating threshold. The Caire ICH software is a deep-learning tool with a single convolutional neural network and a long short-term memory mechanism that provides information regarding the presence or absence of an intracranial hemorrhage, the subtype(s) of the hemorrhage detected, the full range of slices where the hemorrhage exists, and the four slices on which the hemorrhage can be best viewed. 

Performance assessment

We assessed reader performance using inter-reader agreement, accuracy, specificity, and sensitivity. We calculated confidence intervals for each metric using Clopper-Pearson exact confidence intervals. A fourth physician trained in reading NCCT (G.N.) reviewed NCCT scans that both the radiologist aided by the AI system and the AI system missed.

## Results

Cohort characteristics

**Figure 1:**
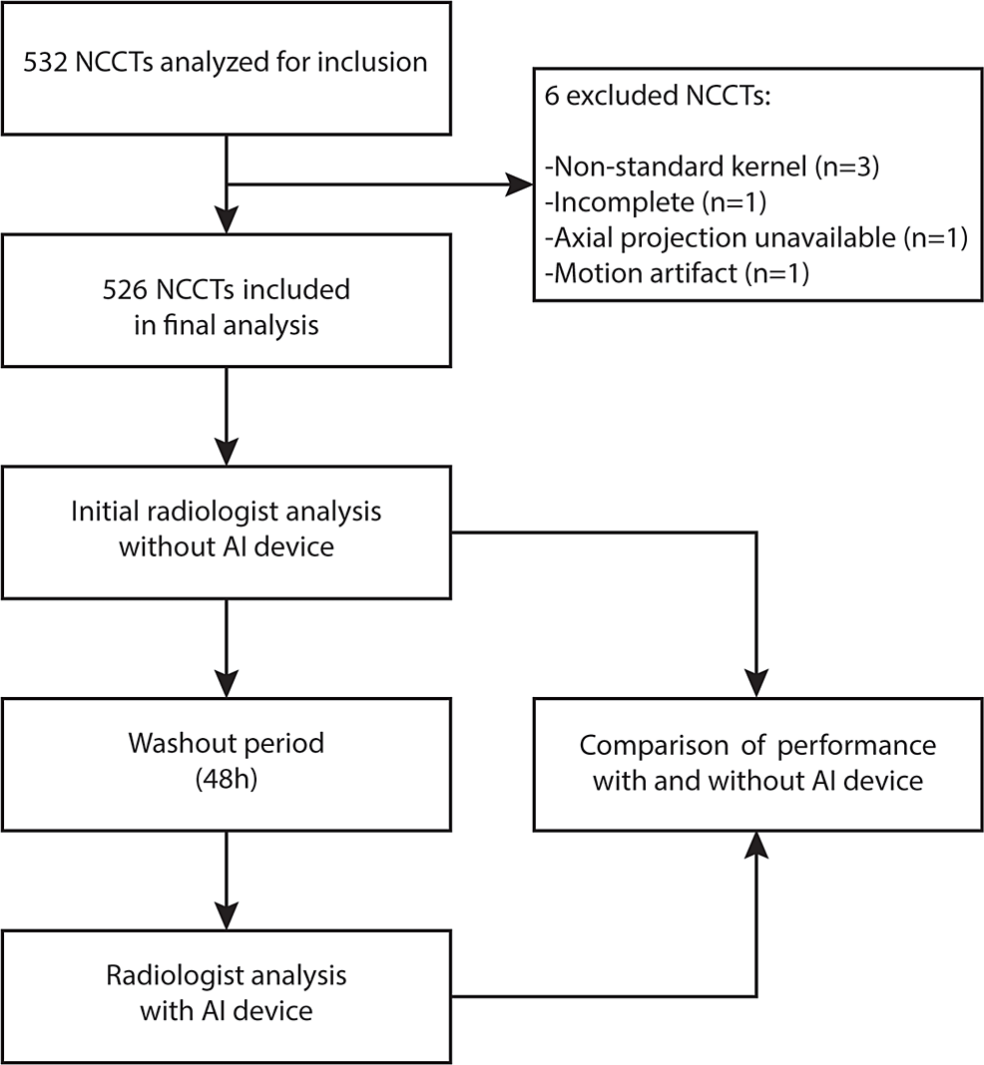
Study design workflow.

We included 526 NCCTs in the final analysis from the initial 600 scans, as summarized in Figure 1. Of all the included NCCTs, 74.3% (n=391) of the NCCTs were determined to have an intracranial hemorrhage. Overall, 37.3% (n=196) featured an IPH, 38.8% (n=204) featured an SDH, 29.8% (n=157) featured an SAH, 22.6% (n=119) featured an IVH, and 4.4% (n=23) featured an EDH. Overall, patients with an intracranial hemorrhage were older (mean of 68.7 years old) and more likely to be male (57.5%), as summarized in Table 1.

**Table 1:** Patient demographics. ICH: intracranial hemorrhage; mm: millimeter.

	ICH Present (n=391)	No ICH (n=135)
Age		
18-20	3	0
20-40	33	23
40-60	71	48
60-80	155	53
>80	108	8
Gender		
Male	225	57
Female	166	78
Scanner manufacturer		
Siemens	213	118
Toshiba	72	5
GE Medical Systems	98	14
Phillips	12	2
Hemorrhage subtype (*individual scans may have multiple subtypes)		
Epidural	23	–
Subdural	204	–
Subarachnoid	157	–
Intraparenchymal	196	–
Intraventricular	119	–
Slice thickness (mm)		
1.5–3 mm (n=518)	383	135
>3 mm (n=8)	8	0

Initial reader performance metrics

Three radiologists labeled each image using the labeling platform. Initial reader agreement was measured to be, on average, 82.76%. We found that the initial performance for indicating the presence of an ICH had an average accuracy of 87.70% (95% confidence interval [CI]: 74.38-100%), a sensitivity of 89.86% (95% CI: 69.19-100%), and a specificity of 81.48% (95% CI: 47.40-100%) (Tables 2-5).

**Table 2:** Average radiologist performance metrics on intracranial hemorrhage detection. *Statistically significant at p<0.05. CI: confidence interval.

Performance metric	Initial performance average (95% CI)	AI-augmented performance average (95% CI)	Hypothesis testing results*
Accuracy	87.70% (74.38–100%)	93.85% (78.14–100%)	p=0.0095
Sensitivity	89.86% (69.19–100%)	94.46% (73.30–100%)	p=0.1425
Specificity	81.48% (47.40–100%)	92.10% (75.50–100%)	p=0.1260
Inter-reader agreement	82.76% (69.98–95.54%)	88.47% (74.53–100%)	p=0.1044

**Table 3:** Individual reader accuracy results for initial read and second read post-washout period aided by the artificial intelligence system. CI: confidence interval.

Reader	Initial accuracy (95% CI)	AI-augmented accuracy (95% CI)
1	81.56% (77.98–84.78%)	86.69% (83.49–89.48%)
2	90.46% (​​87.61–92.83%)	96.20% (94.19–97.66%)
3	91.44% (88.72–93.69%)	94.31% (91.97–96.13%)

**Table 4:** Individual reader sensitivity results for initial read and second read post-washout period aided by the artificial intelligence system. CI: confidence interval.

Reader	Initial sensitivity (95% CI)	AI-augmented sensitivity (95% CI)
1	81.33% (77.11–85.07%)	84.65% (80.69–88.08%)
2	98.46% (96.67–99.43%)	100.00% (99.06–100.00%)
3	90.28% (86.90–93.03%)	98.47% (96.70–99.44%)

**Table 5:** Individual reader specificity results for initial read and second read post-washout period aided by the artificial intelligence system.

Reader	Initial specificity (95% confidence interval)	AI-augmented specificity (95% confidence interval)
1	82.22% (74.71–88.26%)	92.59% (86.80–96.39%)
2	67.41% (58.81–75.22%)	85.19% (78.05–90.71%)
3	94.81% (89.61–97.89%)	82.22% (74.71–88.26%)

The ability to accurately determine the subtype ranged from 79.78% (95% CI: 59.16-100%) for identifying SAH to 94.55% (95% CI: 89.10-100%) for EDH. Similarly, we found the sensitivity and specificity of assigning a particular ICH subtype to an NCCT to vary by subtype (Tables 6-8).

**Table 6:** Average radiologist accuracy performance on intracranial hemorrhage subtype detection. *Statistically significant at p<0.05. CI: confidence interval.

Hemorrhage subtype	Initial accuracy average (95% CI)	AI-augmented accuracy average (95% CI)	Hypothesis testing results*
Subdural	85.30% (67.09–100%)	92.90% (77.90–100%)	p=0.0255
Subarachnoid	79.78% (59.16–100%)	91.20% (68.79–100%)	p=0.0036
Intraparenchymal	89.35% (83.18–95.53%)	94.93% (86.53–100%)	p=0.0142
Intraventricular	89.35% (76.94–100%)	94.61% (79.80–100%)	p=0.0464
Epidural	94.55% (89.10–100%)	96.20% (89.05–100%)	p=0.0865

**Table 7:** Reader sensitivity results for initial read and second read post-washout period aided by the artificial intelligence system for each hemorrhage subtype. *Statistically significant at p<0.05. CI: confidence interval.

Hemorrhage subtype	Initial sensitivity average (95% CI)	AI-augmented sensitivity average (95% CI)	Hypothesis testing results*
Subdural	68.14% (13.4–100%)	87.90% (46.14–100%)	p=0.0432
Subarachnoid	54.56% (10.00–99.1%)	83.22% (26.94–100%)	p=0.0212
Intraparenchymal	75.85% (60.62–91.08%)	90.30% (80.30–100%)	p=0.0435
Intraventricular	55.46% (0–100%)	82.35% (8.22–100%)	p=0.0302
Epidural	47.83% (29.12–66.53%)	78.26% (28.77–100%)	p=0.0728

**Table 8:** Reader specificity results for initial read and second read post-washout period aided by the artificial intelligence system for each hemorrhage subtype. *Statistically significant at p<0.05. CI: confidence interval.

Hemorrhage subtype	Initial specificity average (95% CI)	AI-augmented specificity average (95% CI)	Hypothesis testing results*
Subdural	96.17% (90.33–100%)	96.07% (93.15–98.99%)	p=0.8928
Subarachnoid	90.51% (73.21–100%)	94.58% (84.80–100%)	p=0.1662
Intraparenchymal	97.37% (93.03–100%)	96.97% (91.29–100%)	p=0.6595
Intraventricular	99.26% (97.06–100%)	98.20% (94.04–100%)	p=0.1447
Epidural	96.69% (91.36–100%)	97.02% (91.79–100%)	p=0.4975

AI enhanced performance metrics

After a washout period, the same cohort of radiologists labeled the head NCCTs with the AI model's guidance (Figure 1). We found the cohort to have an increase in their agreement regarding the presence of an ICH by 5.71%. Further, we found their average accuracy of identifying an ICH significantly improved by 6.15% to 93.85% (95% CI: 78.14-100%) (p=0.0095). The cohort’s sensitivity and specificity were also found to be increased by 4.6% to 94.46% (95% CI: 73.30-100%) and 10.62% to 92.10% (95% CI: 75.50-100%), though these changes were not statistically significant (Tables 2-5).

We also observed the cohort improve their ability to identify an ICH subtype correctly. Notably, the accuracy for correctly identifying a subarachnoid increased by 11.42% to 91.20% (95% CI: 68.79-100%) (p=0.0036), and the accuracy for correctly identifying subdural hemorrhages increased by 7.6% (95% CI: 77.90-100%) (p = 0.0255) (Tables 6-8).

As our sample was enriched for imaging with intracranial hemorrhages to ensure the testing of the algorithm included a wide diversity of pathology. With regards to the impact this has on the generalization of metrics if such an algorithm was evaluated outside of this research context, we calculated the accuracy of the reviewers assuming a 2% ICH prevalence in a cohort of NCCT scans [18]. The average radiologist's accuracy would be 81.65% (95% CI: 78.07% to 84.86%) prior to software assistance. The radiologist's accuracy would increase to 92.57% (95% CI: 89.99% to 94.67%) following AI assistance.

Expert review of missed scans

**Figure 2:**
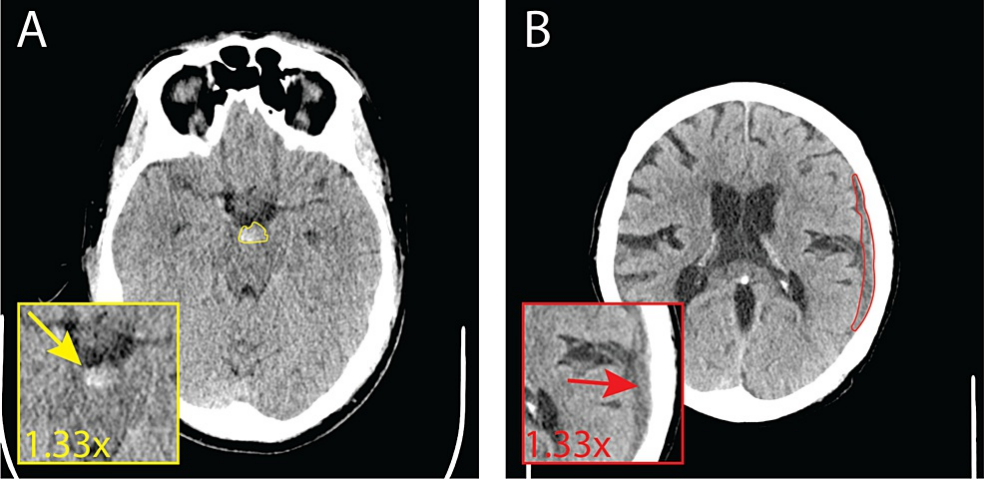
Examples of scans initially missed by radiologists that were correctly predicted with the Caire ICH system. (A) A perimesencephalic subarachnoid hemorrhage (yellow arrow) and (B) a chronic convexity subdural hemorrhage (red arrow).

All three radiologists missed seven out of the included 526 scans, and two of the three radiologists missed 29 of the total included scans. At least one radiologist was able to detect the hemorrhage that they were unable to without the AI in 65 scans. The AI model's output caused two radiologists to identify a hemorrhage in 15 initially missed scans correctly. These scans were reviewed and characterized by a neurologist, and two examples are shown in Figure 2.

**Figure 3:**
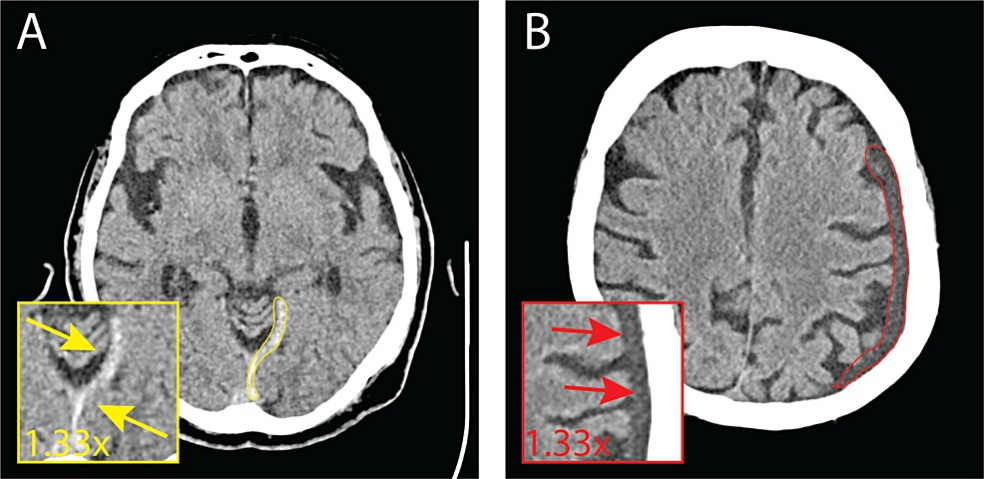
Examples of scans missed by radiologists and the Caire ICH system. (A) An acute tentorial subdural hemorrhage (yellow arrow) and (B) a chronic convexity subdural hemorrhage (red arrow).

Of the 526 scans, 65 individual scans positive for an ICH were missed by at least one radiologist when aided by the AI device (21.6 false negatives and 10.7 false positives on average). Of the total missed scans, the AI system only missed four. Examples of missed cases are illustrated in Figure 3.

## Discussion

This study shows that a board-certified radiologist’s performance for detecting and sub-typing an intracranial hemorrhage can be enhanced when coupled with the Caire ICH artificial intelligence system.

The number of acquired CT examinations requiring radiologist interpretation is growing at a rate that outpaces the growth of the radiologist population, which introduces concerns surrounding the presence of errors in imaging-based diagnoses, particularly for pathologies where misdiagnoses may lead to delays in time-sensitive treatment [3,19-21]. Several studies have evaluated retrospective records of board-certified radiologists and estimated error rates and discrepancies of 3-5% [18,22]. A multi-year, multi-center study previously showed that 5.4% of all patients admitted with a non-traumatic subarachnoid hemorrhage had a previous visit to the emergency room where the subarachnoid hemorrhage was missed [23].

This study implies that future clinical workflows may see AI be used in an adjunct capacity to improve interpretations of CT scans by helping call radiologists' attention to findings that may be overlooked. It enhances inter-reader agreement, the gold standard metric, but also improves an individual physician’s accuracy and sensitivity to diagnose an ICH. Further, the observed increase in performance for sub-typing the detected hemorrhage is potentially beneficial as a patient’s prognosis and management are guided not just by the detection of a hemorrhage but by the subtype identified [14-15].

Finally, diagnostic errors that lead to patient harm are the most common cause of malpractice suits against radiologists [24]. By improving radiologist accuracy in clinical practice, the system studied here might reduce the risk of costly malpractice suits by ensuring that ICH findings are not missed and patients are treated in a timely manner. Further, it is possible that AI systems can increase both the turnaround time of studies with urgent findings and the general speed of interpretation, and future prospective studies evaluating these claims will be useful.

Limitations

There are several limitations to this work. Notably, while we provided the NCCT to readers, no patient history or clinical context was provided. All studies were presented similarly in axial projections and with similar urgency. The lack of a clinical cue may have reduced radiologist performance. Additionally, none of the readers were neuroradiologists, so the readers were more likely to benefit from software assistance. Other notable limitations include the small number of readers and the disproportionately high prevalence of ICH in the dataset that does not correlate with its natural incidence in real-life clinical contexts. We also did not specifically assess the software’s performance concerning intracranial hemorrhage mimics (e.g., brain tumors). The short washout period before readers reviewed the same scan aided by AI may have contributed to an increased diagnostic accuracy with software assistance. However, the randomization of scan presentations during each review aimed to mitigate that effect. Significantly, the same cohort of radiologists that established the ground truth through either unanimous or majority consensus participated in the testing sessions, possibly introducing memory bias to the results that were not directly accounted for by our methodological design. Though this is a limitation of our methodology, it is congruent with prior deep-learning strategies [25,26]. Finally, the radiologists interpreted these scans on different personal computers through a web-based DICOM viewer that emulated PACS functionality but did not meet the complete ACR guideline recommendations for medical imaging monitors.

## Conclusions

With the ever-increasing number of imaging orders, tools that allow radiologists to optimize workflow and minimize the risk of errors and discrepancies become more critical. Here, we present an AI system that can improve the performance and accuracy of emergent image interpretation. This work adds to the evidence that deep-learning strategies may increase the performance of trained radiologists in diagnosing critical findings on head NCCT scans. As we have shown, this strategy may be seamlessly integrated into the radiologists' clinical workflow and potentially expedite the diagnosis and treatment of patients with ICH in resource-rich or resource-limited areas. Future efforts should develop similar models to improve radiologists' diagnostic throughput and performance. Furthermore, further validation of this software in real-world datasets will be essential to solidify its applicability in current clinical practice.
